# Five levels of famine prevention: towards a framework for the twenty‐first century and beyond

**DOI:** 10.1111/disa.12668

**Published:** 2024-11-07

**Authors:** Paul Howe, Merry Fitzpatrick, Daniel Maxwell

**Affiliations:** ^1^ Feinstein International Center, Friedman School of Nutrition Science and Policy Tufts University United States

**Keywords:** climate change, conflict, famine, framework, humanitarian needs, prevention, risk

## Abstract

In recent years, the world has faced a rapid rise in humanitarian needs and an increasing risk of famine. Given the potential threats posed by conflict, climate change, economic shocks, and other issues, it is important to be prepared for the possibility of new crises in the future. Drawing on key informant interviews and a literature review, this paper assesses the state of the art in famine prevention, examining a range of technical and political approaches and analysing emerging lessons. Based on the findings, it identifies five levels of famine prevention: (i) averting famine; (ii) anticipating famine; (iii) reducing famine risks; (iv) altering famine risks; and (v) preventing famine risks. The paper argues that the current focus only partially addresses a relatively narrow set of levels. It concludes that a more comprehensive approach that engages all five levels simultaneously could contribute to a global famine prevention framework for the twenty‐first century and beyond.

## 1 INTRODUCTION

In the past 15 years, there has been a significant reconsideration of the risk of famine globally. While at the start of the twenty‐first century it had been thought that famines were diminishing in number and severity and might soon be eradicated (de Waal, 2015a, 2015b), several recent developments have altered this view. The first is the re‐emergence of famine and near‐famine events. In 2011–12, a famine in Somalia was estimated to have taken the lives of more than 258,000 people (Checchi and Courtland Robinson, 2013). Then, in 2017, there was widespread concern about the risk of ‘four famines’ occurring simultaneously in Nigeria, Somalia, South Sudan, and Yemen, with two of the crises being declared ‘famines’ either at the time or retrospectively. Most recently, in 2024, Sudan is experiencing a famine, and Gaza was identified as at risk of an ‘imminent’ famine (IPC, 2024a, 2024b, p. 2).

The second development is the apparent correspondence of these events to a wider trend of growing humanitarian needs globally. Since its formation in 2014, the Famine Review Committee of the United Nations (UN) has convened some 20 times, with nearly half of these taking place after 2020 (Maxwell, Day, and Hailey, 2023). In roughly the same period, between 2015 and 2023, the number of people requiring humanitarian assistance worldwide increased from 1 in 95 to 1 in 23 people (OCHA, 2022). The long‐term rise in humanitarian needs suggests that the new famines and near‐famines are not isolated, one‐off occurrences but possibly the result of underlying global trends.

The third development is mounting recognition that the trends generating these crises, such as those related to conflict, climate change, and economic issues, are not abating and may augment the risk of famine in the future (OCHA, 2022, 2023), creating greater budgetary pressures for the humanitarian system at a time of declining resources (OCHA Services, n.d.). Conflict, for instance, has been a factor in almost all recent famines, and globally, there has been a rise in climate hazards (OCHA, 2022) and land degradation among the poorest people.

In part because of these realisations, the international community put in place a series of high‐level initiatives focused on famine prevention. In 2020, the United Kingdom government designated a Special Envoy for Famine Prevention and Humanitarian Affairs (Worley, 2022). In 2021, UN Secretary‐General António Guterres created a High‐Level Task Force on Preventing Famine, led by the Emergency Relief Coordinator. Soon after, the G7 (Group of Seven) agreed to a Famine Prevention and Humanitarian Crises Compact. In 2022, the UN appointed a Famine Prevention and Response Coordinator, although the position has now been discontinued. The World Bank has also launched initiatives to address famine better. At the same time, doubts have been raised about the ability and collective commitment of multilateral institutions to take meaningful action on major issues (Gowan, 2023).

Yet, in this context, the world does not have a widely‐agreed framework for famine prevention that can bring coherence to these various initiatives, clarify the diverse range of actions and actors involved, and thereby help to ensure greater effectiveness.

This paper aims to contribute to filling this gap. It draws on a series of key informant interviews and a review of the literature. A total of 59 interviews were conducted with 81 individuals engaged in the humanitarian field. The interviewees included representatives of national and international humanitarian organisations, governments, donor agencies, and academia. They were identified through a snowball sampling methodology and were asked questions in semi‐structured interviews that lasted approximately one hour each. In addition, literature searches of the Web of Science (for peer‐reviewed articles) and Google Scholar (which includes ‘grey’ literature) platforms were undertaken using the general term ‘famine prevention’ with a focus on documents produced since 2011. The literature and interviews were reviewed separately by multiple analysts, each pulling out key topics and trends. The results were compared and discussed in the light of the analysts' collective experiences and expertise.

Based on the findings, we suggest that it is possible to identify five different levels of famine prevention that may serve as the basis for a wider framework. To motivate and elaborate this framework, the paper is divided into five sections. Following this introduction, the second section reviews the findings of the analysis—that is, the lessons, perspectives, and trends drawn from the key informant interviews and literature review. The third section explains the proposed five‐level famine prevention framework which emerges from these results. The fourth section discusses the potential implications of this framework for current approaches to famine prevention. The final section reflects upon the very real challenges of successfully implementing this framework and concludes with suggestions for the way forward.

## 2 FINDINGS

In this section, we examine the findings of the interviews and literature review to understand the current state of the art in famine prevention (Maxwell, Howe, and Fitzpatrick, 2023).^1^ The broad prevention approaches can be grouped into two categories, technical and political, although we will problematise aspects of this distinction. Technical approaches refer to actions aimed at *implementing* the agreed famine prevention priorities of actors. For humanitarian and development actors at the local, national, and international level, these endeavours are often aligned with their established mandates, address issues that they can be reasonably expected to influence, and have impacts on the ground. They may include providing early warning and phase classifications, scaling up anticipatory action and humanitarian responses, carrying out resilience‐ and development‐related projects or policies, and improving coordination efforts.

By contrast, political approaches refer to actions aimed at *changing* the priorities of actors to focus on, or at least accommodate, famine prevention. Given the complex geopolitical interests surrounding conflict and other issues that affect famine, and the multiple contexts that may be involved, these efforts involve a range of actors from the local to the international level, including communities, civil societies, governments, diplomatic corps, non‐governmental organisations (NGOs), UN agencies, and international courts. Examples of political actions include negotiations with governments for humanitarian access in times of conflict, advocacy for greater attention to and resources for neglected crises, changes to policies that marginalise or exclude certain groups (often to the benefit of others), and initiatives to prevent or manage conflict.

While the distinction between technical and political approaches was frequently made implicitly and sometimes explicitly in the literature and interviews, in part because of a perception that humanitarian assistance should be neutral, it is somewhat problematic since the categories cannot be separated neatly. For instance, humanitarian action can be influenced by political considerations when donors allocate resources on the basis of strategic interests. Alternatively, climate action to reduce famine risk may need both political approaches to bring attention to the issue and technical efforts to lower emissions. These considerations will inform some of the recommendations, including the importance of ensuring that technical and political approaches are employed in tandem.

### 2.1 Technical approaches

While there may be many ways to subdivide technical approaches—that is, prevention efforts focused on the implementation of agreed priorities and mandates—we identify six categories that emerged from interviews and the literature: (i) response; (ii) early warning; (iii) anticipatory action; (iv) resilience; (v) the triple (humanitarian–development–peace) nexus; and (vi) development.

#### 2.1.1 Response

In recent decades, there have been major improvements, with some accompanying downsides, in humanitarian responses to emerging famine conditions. One has been agreement on a standardised approach to identifying the level of food insecurity, which has increasingly gained the trust of responders. The Integrated Food Security Phase Classification (IPC) platform, encompassing a consortium led by the UN's Food and Agriculture Organization (FAO), categorises current situations using five different phases, with the fifth one corresponding to ‘Catastrophe/Famine’.^2^ While the IPC has permitted comparison of crises across contexts, its effectiveness can be hampered by data constraints or political reluctance to declare a crisis a famine (Maxwell and Hailey, 2021). Another advance has been a new conceptual understanding of famines as complex systems (Howe, 2010, 2018; Fortnam and Hailey, 2024), although the potential implications for response have not yet been fully explored.

There have also been significant advances in humanitarian action itself, including: the shift to greater use of cash; a recognition of the need for more integrated, multisectoral approaches (Food Security, Health, Nutrition, and WASH Clusters, 2021); the consideration of gender, protection, and accountability to affected populations; the emphasis on community‐based management of acute malnutrition; the introduction of new technologies such as mobile devices and biometrics to enhance these and other interventions (but with attendant risks); and a greater commitment to localisation of responses (Robillard, Atim, and Maxwell, 2021). However, both academic studies (Buchanan‐Smith and Davies, 1995; Maxwell and Hailey, 2020) and interviewees identified triggering timely responses by early warning as one of the largest challenges.

#### 2.1.2 Early warning

Efforts to predict potential crises and intervene before they develop into famines have some of their origins  in the Indian Famine Codes of the late nineteenth century, but modern early warning systems emerged following the Sahelian drought in the mid‐1970s (Walker, 1989). There are now two primary sources of early warning for food security‐related crises at the global level. The first is the Famine Early Warning Systems Network (FEWS NET). Since its founding in 1985, it has grown into a global network that monitors more than 38 countries across the world. The second is the IPC platform. While not formally an early warning system, in classifying the current severity of crises, the IPC also provides projections of the likely levels of food insecurity in three to sixth months. National and local early warning systems have also been established in at‐risk countries such as Ethiopia and Kenya, some of which are linked to the IPC platform, and some of which operate entirely independently.

Broadly speaking, early warning systems have been effective in predicting crises. Reviewing FEWS NET's forecasts in 25 countries, Backer and Billing (2021) found that they were accurate 84 per cent of the time in identifying potential crises (IPC Phase 3 or above). However, the rate fell to only 29 per cent accuracy when specifically forecasting famine (IPC Phase 5)—the lower rate may be explained, though, by Phase 5 forecasts prompting action (even if late) that prevents the situation from developing into a famine. According to Maxwell and Hailey (2020) and interviews conducted for this study, key remaining challenges include: the difficulty of dealing with uncertainty; the abundance of sometimes contradictory information; the politicisation and manipulation of findings; concern about sharing data; and the need for real time monitoring.

#### 2.1.3 Anticipatory action

Anticipatory action refers to providing humanitarian assistance, often in the form of cash and other mitigative interventions, such as the supply of drought‐resistant seeds and livestock protection, based on a forecast, rather than in response to an ongoing crisis. It can be initiated either by triggers or scenarios, which are frequently used with index‐based insurance or shock‐responsive safety nets (Chantarat et al., 2009; Wilkinson et al., 2018; Gentilini et al., 2020). For example, the Hunger Safety Net Programme in Kenya expands horizontally (increasing the number of people reached) and vertically (increasing the size of cash transfers) when a drought threshold is crossed.

Flexibility for anticipatory action has also been built into funding sources through crisis modifiers (Charters, 2015), the Central Emergency Response Fund (Pichon, 2019), the Rapid Response Mechanism of the World Food Programme (WFP) and the United Nations Children's Fund, the United States Agency for International Development (USAID)'s 10 per cent variance that is permitted to accommodate changing circumstances, and a commitment to ‘no regrets programming’ (Lentz and Maxwell, 2022). The latter reflects a calculation, in the face of uncertainty, that the benefits of providing timely assistance at scale, especially if supportive of livelihoods, outweigh the dangers of inadvertently expending resources on a crisis that may not fully materialise (Maxwell and Hailey, 2020; Weingärtner, Pforr, and Wilkinson, 2020).

In practice, according to interviews conducted for this study, the greatest challenges in implementing anticipatory action include: inadequate financing for this approach; its primary relevance to climate rather than conflict hazards; the difficulty of reaching those most in need, who are often in inaccessible areas; and risk aversion by donors and agencies in terms of expending scarce resources early on situations that they cannot be certain will materialise into major crises.

#### 2.1.4 Resilience

Resilience is often understood as creating a link between humanitarian assistance and development to help communities to withstand shocks and continue to progress with development (Barrett and Constas, 2014; Catley, 2017; Maxwell et al., 2017; Dahal et al., 2018). Examining early action and resilience investments in Ethiopia, Kenya, and Somalia, Cabot Venton et al. (2012) and Cabot Venton (2018) found them to yield substantial returns on investment. Many national governments and agencies are undertaking resilience efforts, ranging from Somalia's investments after the famine in 2011–12, to the African Union's resilience strategy to adapt to climate change, to the World Bank's greater engagement in famine and food insecurity issues in contexts of instability. Some authors (see, for example, Scott‐Smith, 2018), however, problematise the term's conceptual ambiguity and its consequent concealment of divergent perspectives, stymying clearer thought and necessary debate. It has also been criticised for being a depoliticised term that focuses on technical aspects and decentres issues of power relations, marginalisation, and justice that are inherent but not always foregrounded in discussions of resilience (Mikulewicz, 2019).

In the interviews, one criticism of resilience was that it has value in contexts of climatic shocks but is less useful in describing the actions that should be taken in situations involving the risk of conflict. Interviewees also expressed frustration that ‘resilience’ has become its own ‘silo’ rather than facilitating the integration of humanitarian and development efforts.

#### 2.1.5 Humanitarian–development–peace nexus

The triple nexus attempts to integrate peace approaches into humanitarian and development efforts, promoting strategies that meet immediate needs while also addressing root causes (CRS, CAFOD, and Caritas Australia, 2016; OECD, 2024). It therefore partially brings together both technical and political approaches.^3^ The possibilities and challenges of this initiative have been discussed increasingly (Kittaneh and Stolk, 2018; Howe, 2019; DuBois, 2020; Fitzpatrick et al., 2021). Some see the potential to enhance coordination in a truly joined‐up manner, whereas others, particularly actors like Médecins Sans Frontières which attempt to adhere strictly to the humanitarian principle of independence, are wary of the instrumentalisation of humanitarian assistance in the service of larger political aims (Belliveau, 2021, as cited in Fitzpatrick et al., 2021). Although some progress has been made on its implementation (FAO, 2021; UNICEF, 2021; ALNAP, 2023), there is a lack of consensus on the value of the nexus in practice (Fitzpatrick et al., 2021). Interviewees either did not focus on the issue or suggested that it may be just a semantic rebranding of resilience to tap into funds earmarked for nexus programming rather than a substantively different approach.

#### 2.1.6 Development

Most famine prevention efforts implicitly concentrate on relatively short time frames. However, Sen (1981, 2000) clarified that famine often results from and reinforces underdevelopment, especially for marginalised groups. Part of a wider paradigmatic shift from viewing development in strictly economic terms to a more multidimensional capabilities approach (ul Haq, 1999), his work has nevertheless been criticised for not fully analysing the role of power in creating and perpetuating underdevelopment (Navarro, 2000). Taking a more planetary approach, Sachs (2015) argues that development must be understood as the outcome of interacting economic, environmental, social, and political systems maintained in a sustainable, equitable manner.

These approaches informed the UN's 17 Sustainable Development Goals (SDGs) (United Nations General Assembly, 2015), which through their focus on issues such as health, education, hunger, climate, and peace contribute to trends that reduce the risk of famine (de Waal, 2018b). Although not mentioned frequently, some interviewees did identify development as the ultimate famine prevention strategy. The occurrence of famine was explained as ‘a failure of development’, requiring more holistic, long‐term approaches and greater political will.

Looking across the interviews, humanitarian key informants concentrated much more on shorter‐term measures such as response and early warning than on medium‐ or longer‐term ones such as resilience, the triple nexus, and development. This short‐term mindset may understandably reflect the mandate, experience, and principles of humanitarians. Yet, it may also suggest a narrower conception of the range of potential solutions, less patience with and trust in the effectiveness of longer‐term approaches, and relatively limited engagement with those focused on these issues.

### 2.2 Political approaches

Political approaches—that is, actions that aim to shift or expand the priorities of actors—have taken on growing importance because of the increasing role of conflict in recent crises, such as in Somalia in 2011–12, South Sudan in 2017, Ethiopia in 2022, and Gaza and Sudan in 2024, and the re‐emergence of sieges, mass starvation, and the denial of humanitarian access as tactics of war. There is also much greater recognition of the role of power relations, persistent inequalities, and marginalisation in creating vulnerability to famine locally and nationally. Even though the distinctions are not always clear‐cut, it can be helpful to divide political efforts into four broad categories to highlight the types of approaches that can be employed in this area and their relationship to crisis events: (i) humanitarian advocacy and diplomacy; (ii) political advocacy and diplomacy; (iii) accountability mechanisms; and (iv) political will.

#### 2.2.1 Humanitarian advocacy and diplomacy

Although many definitions have been proposed for ‘humanitarian diplomacy’ (Régnier, 2011), it can be seen as encompassing advocacy efforts, partnerships, and negotiations related to humanitarian responses (Turunen, 2020). In this paper, we will refer to advocacy and partnership efforts as ‘humanitarian advocacy’ and negotiations as ‘humanitarian diplomacy’. Undertaken by civil society, governments, the UN, international non‐governmental organisations (INGOs), local NGOs, and affected populations, humanitarian advocacy is most often employed when crises are already largely in motion and can spotlight human rights protection, respect for international humanitarian law (IHL), and the need to garner attention and resources for neglected crises and especially the groups most affected. Recent examples include advocacy to: prevent the targeting of hospitals, water systems, and other infrastructure needed for survival in Gaza; establish cross‐border humanitarian assistance during the war in Syria, leading to UN Security Council (UNSC) Resolution 2165 of 2014; and mobilise attention and resources to prevent the ‘four famines’ in Nigeria, Somalia, South Sudan, and Yemen in 2017. These initiatives often attempt to build coalitions of partners that can be more persuasive and impactful collectively (OCHA, 2021). But the challenges in practice highlight the difficulty of undertaking these approaches.

Humanitarian diplomacy focuses on negotiation and is ‘a strategy of influence implying interaction with a wide variety of players for an exclusively humanitarian purpose’ (Harroff‐Tavel, 2006, p. 2). These efforts may centre on securing humanitarian access (especially in conflict settings) or addressing bureaucratic impediments and may require both high‐level diplomacy and on‐the‐ground actions (Minear and Smith, 2007). Negotiation for humanitarian access is highly political since it may involve trying to shift or expand the priorities of the parties to a conflict to permit humanitarian assistance to areas where they may have competing interests. Recent examples include the UN negotiating access to gang‐controlled Cité Soleil in Haiti and securing the Black Sea Grain Initiative to permit the export of Ukrainian grain (OCHA, 2022). The International Committee of the Red Cross (ICRC) has also been one of the most effective actors in securing access across a range of complex contexts, particularly through adherence to the principles of neutrality and independence.

Interviewees saw these humanitarian advocacy and diplomacy efforts as having achieved mixed results so far. Although there have been cases of success in advocacy, as mentioned above, they cited the tension between humanitarianism and political engagement as one of the serious limitations to speaking out more strongly. While advocacy around the ‘four famines’ in 2017 mobilised attention and resources, in many other cases it has been difficult to obtain the required funding at scale and in a timely manner. In regards to negotiations, humanitarian interviewees again expressed frustration, referencing recent crises in Ethiopia, South Sudan, and Sudan as examples where access was challenging. They attributed these limitations to the complex interests involved, the balance between public and private diplomacy, and the gaps in the system in terms of trained and engaged actors.

#### 2.2.2 Political advocacy and diplomacy

While humanitarian advocacy and diplomacy is a more reactive process that attempts to create space for a humanitarian response within a challenging context, political advocacy and diplomacy is aimed at changing the context itself. Conflict is acknowledged as a leading cause of food insecurity and famine (FAO, 2017) and can be seen as a key driver of recent crises in Ethiopia, Gaza, and Sudan, and arguably the result of a political process gone awry. When fighting is ongoing, political advocacy may take forms ranging from UNSC resolutions to peaceful protests and other actions that draw attention to the impact of the crisis and call for an end to hostilities. Political advocacy may invoke the humanitarian dimension, but it is distinguished from humanitarian advocacy by its principal focus on changing the broader political context. As demonstrated in relation to the war in Gaza in 2024, civil societies, national governments, and others may take a leading role in these actions, going beyond formal institutions such as the UN (Gowan, 2023).

Political diplomacy focuses on conflict resolution or efforts to negotiate an end to warfare and may involve local or indigenous actors, regional bodies, national governments, or UN entities. Although it is most often employed in contexts of violent conflict—for example, the efforts to end the fighting in Gaza and Sudan in 2024—it is also relevant in situations of marginalisation of specific groups that contribute to famine risk. Recent discussions of conflict prevention, or altering political dynamics before they lead to conflict, have concentrated on ‘pathways for peace’ and sustaining peace (United Nations and World Bank, 2018), recognising the role that grievance, inequality, and exclusion can play in generating violent conflict, and identifying processes and actions to address them.

A few humanitarian interviewees mentioned conflict prevention as a critical factor in famine prevention, but they also expressed uncertainty over how to tackle it in a meaningful manner, perhaps reflecting their relative lack of engagement and comfort with these approaches. It may also be symptomatic of frustration with the seeming intractability of protracted conflicts, such as in South Sudan, or deeply entrenched political positions, as in Gaza, in which multiple attempts to broker a ceasefire have faltered.^4^

#### 2.2.3 Accountability mechanisms

Accountability mechanisms refer to the range of means of holding actors responsible for the creation of famine conditions. They fall under the category of political approaches because they represent an attempt to deter actors from making famine creation a priority or, through the application of the mechanisms, to assign culpability and associated punitive measures to prevent them from making it a priority in the future. There are two broad means of accountability: legal; and sociopolitical.

With precedents in ancient history and origins in the 1864 Geneva Convention, the legal framework that creates accountability for the use of starvation as a weapon of war (Conley et al., 2022) has evolved over time and has two primary instruments: IHL; and international criminal law (ICL). IHL prohibits the starvation of civilians as a method of warfare and the attack or destruction of objects indispensable to the survival of civilian populations, whereas ICL, which identifies the intentional use of starvation as a war crime, strengthens the enforcement of IHL. UNSC Resolution 2417 provides a channel for FAO and WFP to report on situations of hunger and conflict and empowers the UNSC to refer cases to the International Criminal Court (ICC). The recent cases brought before the ICC and the International Court of Justice (ICJ; which focuses on states) referencing the humanitarian situation in Gaza provide an example of this use of accountability, but also point to the challenges faced. Accountability through the utilisation of these legal instruments to prosecute perpetrators differs from invoking IHL in humanitarian advocacy, where the emphasis is on highlighting the disparity between actions and legal standards.

In line with academic studies (de Waal, 2018a; Conley et al., 2022), interviewees viewed legal accountability mechanisms as having significant potential to deter famine creation but expressed concern about the limited, incremental contributions they have made in practice so far, despite their long history. They attributed the limitations to: the sensitive trade‐off between preserving humanitarian operations that require the assent of parties to a conflict and being perceived as part of accountability efforts that could implicate those parties; the gaps in coordination between the UNSC and humanitarian agencies in the field; the limited enforcement of accountability in practice; and the perverse incentives created for potential perpetrators to limit humanitarian presence—for example, in Ethiopia or South Sudan—if they suspect an intention to gather evidence for subsequent prosecution of war crimes (Maxwell and Hailey, 2021). Yet, a smaller number also argued for the need to take a longer‐term perspective, asserting that accountability frameworks, when enforced, change priorities over time as part of a cumulative effect.

In regard to sociopolitical accountability for famine, there are three broad types. The first relates to the social contract in which a government makes a commitment—an anti‐famine contract—to prevent famine and can be held accountable for upholding it (Sen, 1983, 2000; de Waal, 1996). Democracies with a free press are more likely to have an anti‐famine contract, although there are exceptions—see Keen (1994) on democratic Sudan—and it is often politically marginalised groups within a country that are most vulnerable (Majid and McDowell, 2012).

The second type of sociopolitical accountability relates to situations in which hunger and famine lead to violent conflict aimed at triggering changes in policies or regimes. Recent studies (Brück, Justino, and Martin‐Shields, 2017; FAO, 2017; Brück and d'Errico, 2019) suggest that hunger, especially in the form of rising food prices and competition for resources, can exacerbate tensions and contribute to conflict, although the understanding of these relationships is incomplete.

The third type of sociopolitical accountability is the use of other types of external leverage—withholding development assistance, reducing security partnerships, applying sanctions, or causing embarrassment through public discussion of issues—to hold governments and other actors to account for starvation. In some cases, however, these levers may be employed to support geopolitical interests and contribute to famine, as in Bangladesh in 1974 when United States' assistance was withheld at a time of crisis in a bid to influence government policy.

Of these three forms of sociopolitical accountability, interviewees focused primarily on the third—there was limited mention of the social contract or famine triggering policy or regime changes. In considering leverage, often as part of discussions of humanitarian diplomacy, some expressed frustration with the ineffectiveness of the tools and the unwillingness to utilise them, whereas others cautioned about the need to wield them carefully to avoid inadvertently making complex situations more difficult. The relative lack of focus on the social contract or societal change may again reflect the narrow expertise and mandate of humanitarians, as well as a more limited view of how to prevent famine.

#### 2.2.4 Political will

In many ways, the creation of famine is a political act, and so is its prevention. Political will is critical for all of the technical and political approaches described. In practice it has proven elusive and difficult to maintain for at least two reasons. First, relevant actors may have higher priorities to which famine prevention is subordinated (Howe, 2006). Taking a historical example, in Greece during the Second World War of 1939–45, the British blockade was kept in place despite famine warnings because of the larger goal of choking off supplies to the occupying German Army. Second, numerous actors may be involved with different priorities, making alignment difficult—as exemplified by the challenges in achieving global climate action (Sachs, 2015), which is relevant to reducing famine risk. In interviews, political will was cited as a critical factor in famine prevention, but mainly in the context of implementing more proactive efforts towards anticipatory action and resilience rather than tackling longer‐term trends that affect famine risk.

## 3 FIVE LEVELS OF FAMINE PREVENTION

Looking across these findings, it is possible to see that the various technical and political approaches have different stances towards famine prevention in terms of addressing symptoms or deep causes and make contributions at different temporal and spatial scales. For instance, some actions like humanitarian response or humanitarian diplomacy focus on an ongoing crisis in a specific location, whereas others relate to longer‐term trends such as conflict or climate change at the global level. Recognising these different yet complementary approaches, we have developed a broader, more comprehensive framework that attempts to clarify how these diverse efforts can work together to prevent famine more effectively.

**FIGURE 1:**
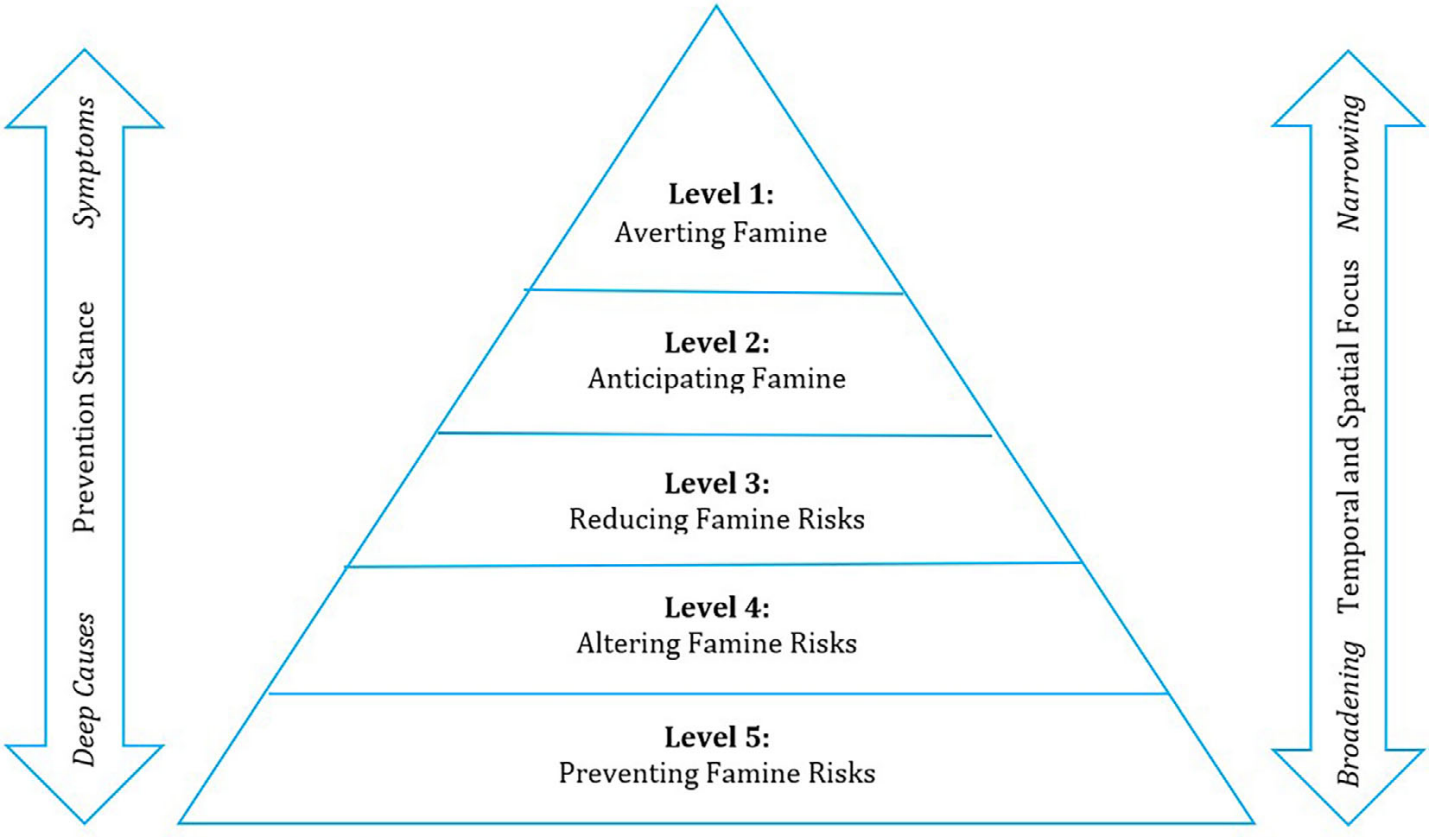
Five levels of famine prevention **Source:** authors.

We identify five levels of famine prevention: (i) averting famine, or reacting to existing crises; (ii) anticipating famine, or responding early to emerging crises; (iii) reducing famine risks, or focusing on areas at high risk of crises; (iv) altering famine risks, or influencing the current trends that generate crises; and (v) preventing famine risks, or shaping integrated trends to eliminate proactively future crises (see Figure 1). At all of these levels, technical and political approaches need to be used in tandem; as discussed, technical approaches often have political dimensions and vice‐versa. The framework should therefore be understood as a way of identifying key factors but not as a limiting structure that prevents more flexible and fluid interpretations and applications.

To help illustrate the employment of the framework, we will describe each level and explain how it would apply to Somalia in 2011–12, the largest famine in the twenty‐first century, and in 2017, when a similar crisis in the country led to far fewer deaths.

### 3.1 Level 1: averting famine

Level 1 prevention is characterised by urgent reactive efforts to prevent existing crises from tipping over into famines.^5^ Both technical and political approaches are critical. On the technical side, IPC assessments constitute an important approach for determining the current status of areas experiencing crises and identifying the risk of famine, whereas humanitarian responses—whether in the form of cash, food, special nutritional products, emergency health and water, sanitation, and hygiene services, or non‐food‐items—may be required to assist affected communities in addressing needs created by the crises. National governments often lead IPC classification analyses—although sometimes problematically because of their stake in the outcomes—with international support (such as from FAO, WFP, and INGOs), while affected communities are at the forefront in responding, supported by the national government, civil society organisations, the private sector, the diaspora, and the international humanitarian system (such as UN agencies, INGOs, and donors).

On the political side, humanitarian advocacy and diplomacy is required to draw attention to IHL in situations of conflict, ensure sufficient resources are allocated, and negotiate access where necessary. It is most effective when used in conjunction with accountability mechanisms. In theory, the explicit or implicit threat of accountability—in the form of reference to legal instruments or sociopolitical levers—can help to shift the calculus for actors and create possibilities for changes in priorities that may allow access for humanitarian assistance. The key actors involved will depend on the situation. Local, national, and international humanitarian entities or civil societies might engage in advocacy (such as UN agencies, INGOs, and local grassroots organisations) or negotiations (such as the United Nations Office for the Coordination of Humanitarian Affairs and the ICRC) with states or nonstate armed groups. These activities may also involve higher‐level bodies such as the UNSC and international courts.

The famine in Somalia 2011–12 took place in the context of La Niña droughts, an ongoing conflict between Al‐Shabaab and the government and its allies, and an extreme global food price spike. As an example of Level 1 approaches and how they work in tandem, the technical IPC declaration of famine in August 2011 triggered a massive increase in funding for the crisis. But the delayed humanitarian response had been impeded by counterterrorism restrictions that put agencies operating in Al‐Shabaab‐controlled areas in legal jeopardy if aid was diverted and ended up in the hands of this Sunni Islamist group. A behind‐the‐scenes humanitarian advocacy effort to work out a humanitarian exemption to counterterrorism regulations was a political approach that facilitated humanitarian agencies to undertake a technical scale‐up, including to Al‐Shabaab areas (Maxwell and Majid, 2016). However, by that time, famine mortality had already peaked, eventually leading to more than 258,000 deaths (Checchi and Courtland Robinson, 2013). By contrast, in 2017, in part because of lessons learned, the focus was more on Level 2 responses, resulting in a timelier technical scale‐up that prevented excess mortality from reaching the massive total of 2011–12.

### 3.2 Level 2: anticipating famine

Level 2 involves shifting from a purely reactive to an anticipatory approach. In recent years, the global aid community has made more deliberate efforts to engage in Level 2 prevention. Temporally, the focus is on potential famines in the form of emerging crises where shocks have affected vulnerable areas and an emergency is developing but is not yet at high risk of tipping over into famine. Spatially, these emerging hotspots and any related crises that may influence their development (such as the impact of the Ukraine war on crises on the African continent) would likely occur in a wider area than those at Level 1 since this set of potential crises is larger than the subset that are at high risk of tipping over into famine. While Level 2 is most closely associated with emerging crises, even during Level 1 humanitarian responses to existing crises, especially in situations of a rapid scale‐up, there is also a need for an ‘anticipatory approach’—thinking about how actions taken will affect drivers and outcomes in three to six months.

On the technical side, early warning systems linked to anticipatory humanitarian action mechanisms (such as cash transfers triggered as part of a shock‐responsive social protection system) constitute the main current approach. Yet, challenges remain in ensuring that early warning leads to early action, especially when making the trade‐off between known current needs and future risks in the context of a constantly resource‐scarce operating environment. While these efforts may involve national and international humanitarian actors (such as ministries responsible for shock‐responsive social protection and emergency response, local NGOs, UN agencies, and INGOs), diasporas, and the private sector, at‐risk communities' own equivalents to formalised early warning and anticipatory action are central to Level 2 endeavours.

There has been less focus on the potential political side at this level. It would be reasonable to propose what might be called ‘anticipatory humanitarian advocacy and diplomacy’, which refers to early advocacy initiatives to call attention to the need for action in these emerging or evolving hotspot areas and to initiate proactive negotiations, leveraging accountability mechanisms where required, to ensure the unimpeded delivery of anticipatory humanitarian assistance. It may also entail broad‐based political advocacy and diplomacy to change the ‘context’ of conflict that is generating the crisis. These efforts would draw on many of the political actors engaged in Level 1 and may also include entities focused on peace.

In Somalia in 2011–12, Western donors largely ignored the early warnings of an emerging crisis that were first issued in late 2010 (Hillbruner and Moloney, 2012; Maxwell and Majid, 2016). There were several higher political priorities and considerations at the time: an offensive against Al‐Shabaab; the desire to prevent humanitarian assistance from inadvertently reaching this designated terrorist group; and uncertainty about whether a severe crisis would in fact materialise. At the same time, the struggle for national political power and the role of food aid as a political resource meant that assistance did not reach those in greatest need in rural areas (Jaspars, Majid, and Adan, 2023). Without external support, affected populations relied on social networks including diaspora members (remittances) and extended clans, but groups—such as the Bantu and Rahanweyn with limited networks and which had experienced marginalisation—struggled to survive (Majid and McDowell, 2012; Maxwell et al., 2016).

In 2017, by contrast, the Somalia NGO Consortium engaged early on in a series of political advocacy initiatives drawing on technical early warning and IPC analysis. Although still late, donors responded more quickly than in 2011–12, and the newly functioning national government coordinated responses by international and local humanitarian agencies, the private sector, and the diaspora (DuBois, Harvey, and Taylor, 2018; Clayton, Ibrahim, and Yusuf, 2019). Jaspars, Majid, and Adan (2023) argue that a shift to a more collusive political marketplace was an added factor, as political actors may benefit from avoiding famine as this keeps external resources coming in. They also benefit from maintaining a large group of displaced populations in a state of precarity as a source of cheap and exploitable casual labour.

### 3.3 Level 3: reducing famine risks

Level 3 involves a shift from a focus on crises themselves to areas at higher risk of crises. In practice, this means investing in measures that reduce the vulnerability of communities to famine and decrease the likelihood of locally‐generated shocks or inadequate humanitarian action. It concentrates on areas and marginalised populations that are analysed as at risk of experiencing the confluence of factors that lead to famine and therefore represents a wider set of locations than Level 2 crises.

On the technical side, reducing local famine risks can take two broad forms: (i) decreasing the vulnerability of communities through resilience and triple nexus activities; and (ii) putting in place crisis support mechanisms—such as national government shock‐responsive safety nets, diaspora remittance systems, or international humanitarian assistance—that can supplement community responses. These efforts would centre particularly on marginalised groups and would include conducting a closer, more critical review of structural aspects that foster power inequities that inhibit certain populations from accessing the resources and services necessary to thrive, or even to cope with shocks. They would involve resilience and triple nexus actors for the reduction of vulnerability and social protection and humanitarian experts for crisis support mechanisms.

On the political side, efforts would be directed at shifting priorities away from actions that might lead to crises. For example, they might entail the strengthening of accountability mechanisms in the form of a social contract at the national level to support safety nets and other responses, or political advocacy and diplomacy in the form of conflict resolution and prevention to reduce the risk of initiation or expansion of violence. Efforts would involve taking actions that address structurally‐embedded power relations and the unequal distribution of resources that leave certain groups more vulnerable to the effects of shocks and at greater risk of experiencing famine—and which in turn can contribute to conflict, generating famine risk. Local, national, and international actors with expertise in governance, development, inequalities, and peace would be especially relevant at this level.

In 2011–12, Somalia had gone several decades without a functioning national government, and Al‐Shabaab had launched an offensive on the capital, Mogadishu. There were limited technical investments in resilience and safety nets or a focus on political solutions amid the ‘Global War on Terrorism’. Moreover, some clans had been excluded from access to many resources and occupations for generations and frequently from government services and assistance by gatekeepers. These marginalised groups would not only need to be favoured in humanitarian responses, but to decrease their famine risks, the structures maintaining these inequities would need to be addressed prior to the famine's development. In the end, their marginalisation in both development and humanitarian spheres disproportionately increased the felt effects of the famine and led to higher loss of livelihoods and ultimately of lives.

By 2017, donors had supported systematic resilience investments against drought (Clayton, Ibrahim, and Yusuf, 2019; Martin, 2019), and the Federal Government of Somalia, along with the Federal Member States, had improved capacity to engage in a coordinating role. Donor agencies already had humanitarian exemptions in place for counterterrorism restrictions. While these factors alone are not responsible for the improved outcomes, they may have helped to reduce vulnerability, at least to famine (Clayton, Ibrahim, and Yusuf, 2019; Martin, 2019). Nevertheless, persistent inequalities and the ongoing marginalisation of certain populations, regardless of geographic location, continue to raise the risk of famine for these populations.

### 3.4 Level 4: altering famine risks

Level 4 prevention alters famine risks by influencing the trends at the global level that lead to these crises. It recognises that shocks and vulnerability result from deeper, broader systemic causes that produce these trends and begins to modify these underlying dynamics. Temporally, since these efforts aim to influence longer‐term trends such as climate change, they may require more time but potentially would also have longer‐lasting benefits. Spatially, they address trends at a global scale, but the emphasis is on countries that may be most affected by them in the future.

The technical approaches would involve the reinforcement of positive trends such as global development—as broadly defined in the 2030 Agenda for Sustainable Development and likely any subsequent frameworks—and the expansion of the global humanitarian system to lower famine risk. The focus would be on enhanced and expanded programmes supported by investment of resources and innovations to increase effectiveness, often in line with country‐specific strategies. It would be technical in the sense of implementing globally‐agreed priorities. But tackling some of the structural inequalities, not just their effects, requires a complementary, coordinated political approach.

On the political side, Level 4 actions entail altering negative or mixed trends such as conflict, climate change, economic volatility, lack of accountability, and persistent inequalities arising from globalisation. Within globalisation, for instance, unequal trading systems that persist, in part, from colonial and neocolonial power imbalances can weaken local governance and economies and should themselves be modified rather than depending only on contributions from wealthier donor countries, providing aid to nations they may have helped make vulnerable through these systems (Rodrik, 2012). Looking across these trends, it would necessitate a major undertaking to shift priorities to ensure that the political will, norms, structures, and enabling environment are in place to align actors to make these deep and profound changes at a level, speed, and sophistication commensurate with the problems. The current context of budget cuts and a general pulling back of the international community (Gowan, 2023) may call for a broader‐based or potentially radically different approach. The relevant actors therefore would have to range from governments and multilateral institutions to civil societies and advocacy groups, with the focus of change, at times, directed inward for wealthier countries, rather than solely towards correcting issues and drivers within more marginalised ones.

While the crises in Somalia in 2011–12 and 2017 can be presented too starkly as contrasting cases of famine and famine prevention, the underlying trends that led to their occurrence and which may contribute to future crises were not fully addressed in either case. For example, it is possible (although not proven) that the increasing frequency of drought in the country is in part associated with manifestations of climate change. In this period, the rate of increase in global greenhouse gas emissions declined in comparison to the first decade of the century, but continued to rise (Olivier and Peters, 2020), while the number of natural hazards globally also continued to increase (OCHA, 2023). Issues of inequality within Somalia and with respect to Somalia within the global community have also not been tackled. These risks would need to be addressed as a Level 4 action to alter the risk of famine in the country and elsewhere, particularly when overlain with conflict and market shocks.

### 3.5 Level 5: preventing famine risks

Level 5 is the most proactive and long‐term approach and widens the focus from altering the individual trends that are leading to the risk of famine to taking a broader, more integrated, and forward‐looking approach to shaping priorities, dynamics, and associated trends that support famine prevention. This broadening relates to looking not only at the trends in Level 4, but also at emerging or potentially extreme versions of trends. Emerging trends might include artificial intelligence (AI), which has the potential to augment or diminish famine risks, while extreme versions of trends such as conflict might involve limited nuclear war which could, among other horrific impacts, induce famine (Xia et al., 2022).

The integration involves seeing these trends and existing ones as an interrelated whole, presenting changing profiles of risk for famine on the planet over time. For instance, we can think of trends in climate, agriculture, population, and famine as ‘knotted’. Climate change may add to the risk of famine by intensifying the number of hazard shocks and creating conditions—through warming, loss of arable land, sea‐level rises, and variations in cropping seasons—that make communities more vulnerable to them (Jägermeyr et al., 2021; Richards, Gauch, and Allwood, 2023). Agriculture is a primary source of climate emissions (Lynch et al., 2021), but production would need to keep up with an increasing, for now, global population that may take different trajectories in different regions during this century (Gu, Andreev, and Dupre, 2021) and may introduce new considerations and complications when levels start to decrease. Likewise, the approach might need to consider the impact of input‐intensive food systems in wealthier countries on the climate and therefore on risks to low‐input rainfall‐dependent agricultural systems in more famine‐prone nations. In this context, actions on climate, agriculture, and population must be considered together and take into account the implications for famine risk.

By looking at trends broadly and seeing these kinds of ‘knots’ that need to be ‘untangled’ in relation to and consideration of each other, forward‐looking proactiveness involves working to address these complexities and actively foreseeing new possible knots of concern—and shaping trends to avoid them pre‐emptively. It is important to emphasise that these broader concerns related to climate change, conflict, agriculture, and population have consequences and importance that go well beyond the risk of famine. However, a holistic famine prevention strategy needs to recognise the relevance of these issues and the stake that famine prevention concerns have in their resolution.

On the technical side, the focus would be on developing an integrated view of these different trends and knots and what they might mean for the risk of famine—both ‘horizontally’ across challenges (such as those pertaining to climate, agriculture, population, and famine), but also ‘vertically’ from the planetary to the local and individual level. It might also entail making investments to address identified risks ahead of time. On the political side, the emphasis would be on establishing famine prevention as a recognised worldwide priority (such as by including it in the successor framework to the SDGs) and taking actions consistent with this integrated and holistic understanding.

For instance, in Somalia, a widely‐accepted political global priority of famine prevention might have led to a more proactive stance towards the emerging crises in both 2011–12 and 2017. Moreover, an integrated analysis may have identified the potential confluence of these trends without the offsetting safety nets at the national or international level in 2011–2 drawing attention to the heightened risk. However, the benefits would be felt most clearly not in the short term, but through the addressing of the interrelated trends that would reduce the risk of famine at the planetary level far into the future. Table 1 summarises these approaches at different levels.

**TABLE 1:** Technical and political approaches at different famine prevention levels.

Level	Name	Description	Technical approaches	Political approaches
1	Averting famine	Reacting to existing crises	IPC classifications. Humanitarian response.	Humanitarian advocacy and diplomacy. Accountability mechanisms.
2	Anticipating famine	Responding early to emerging crises	Early warning. Anticipatory humanitarian action.	Anticipatory humanitarian advocacy and diplomacy. Accountability mechanisms. Political advocacy and diplomacy.
3	Reducing famine risks	Focusing on locations most at risk of crises	Resilience and nexus programming. Establishment of national social protection systems.	Political advocacy and diplomacy. National accountability mechanisms.
4	Altering famine risks	Influencing current trends that generate the risk of crises globally	Reinforcement of positive technical trends (such as sustainable development and humanitarian systems).	Adjustment of negative or mixed political trends (such as climate change, conflict, economic integration, and accountability).
5	Preventing famine risks	Acting on an integrated view of future planetary trends and hazards	Taking a broader, more integrated, and forward‐looking view of trends. Identification of potential planetary knots and hazards associated with those trends.	Establishment of famine prevention as one of the key priorities for humanity. Proactive engagement in wider discussions to shape trends and address knots and hazards.

**Source:** authors.

## 4 DISCUSSION

Placing the findings in this framework helps us to understand the range of possible prevention strategies and clarifies where we have focused current attention and where potential gaps remain. The analysis highlights five broad limitations in current approaches to famine prevention.

### 4.1 Most of the current focus is on the more ‘reactive’ levels of the framework

Current famine prevention efforts primarily address Levels 1 and 2, with limited attempts to engage at Level 3. Almost all of the *Global Humanitarian Overview 2024* requirements of USD 46 billion centre on technical humanitarian response (cf. OCHA, 2023). In many ways, the reduction in famines in the past 30 years is a tribute to the investments in Levels 1 and 2 technical prevention and represents significant progress for humanity. However, in the face of increased risk of conflict, climate hazards, and other drivers, the danger is that this approach addresses the symptoms rather than the causes of the problem and is an incomplete strategy for preventing famines, since the number of Level 1 crises are driven by what happens in Levels 2–5.

As reflected in the interviews and literature, the disconnect may arise because humanitarian practitioners and scholars are in a sense mandated to concentrate on the more reactive levels, whereas the peace and development actors do not see famine prevention as their primary role or even within their remit. This is one reason why a framework that explicitly demonstrates how these efforts are interrelated and interdependent is so important.

### 4.2 Much of the current focus is on technical rather than political approaches

For years, the humanitarian community has invested in technical approaches such as early warning and improved humanitarian action, creating structures from the local to the global level. But no comparable system exists on the political side. As a result, we have seen that there are gaps in the framework with few effective political equivalents to the technical ‘early warning’ and ‘anticipatory action’, much less engagement at Levels 3–5. Yet, even in a global environment that is not currently very amenable, political approaches have the potential for larger impacts with possibly far less financial investment.

As indicated in the interviews, many humanitarians feel that they face genuine operational constraints in raising political issues, perceiving a trade‐off between access and presence and political engagement. In this context, the aim may be to: (i) develop a political ‘system’ that is complementary to the technical one, drawing on actors with the appropriate mandates and capacities (such as civil society and governments in humanitarian advocacy and diplomats in humanitarian negotiation); and (ii) recognise that technical and political approaches are not mutually exclusive, competing undertakings but need to be used skilfully in tandem.

### 4.3 The current focus is on famine as a standalone issue

At Levels 3, 4, and 5, the challenges that need to be addressed become less specifically humanitarian in nature and more far‐ranging issues for the planet, such as conflict, climate change, demographics, and socioeconomic disparities, which have impacts (and potential solutions) that go well beyond just famine prevention. However, the implicit assumption that famine is a humanitarian concern and that issues like development and conflict prevention are not perceived as having a central role in tackling the problem undermines a more comprehensive and effective approach.

Instead, at these levels, famine prevention should be pursued not as a standalone priority, but rather as part of a ‘bundled set’ of urgent priorities for humanity that together prevent the creation of conditions that might lead to famine, but which also benefit humanity more broadly. These actions would not be performed solely or even primarily to prevent famine, but rather famine prevention would be deliberately incorporated into the way that they are undertaken to ensure that the entire set of priorities is achieved. For instance, if climate action was implemented without consideration of the impact on agriculture, or conflict was reduced but without proscribing the use of hunger as a weapon of war, the wider set of priorities that includes famine prevention would not be met.

### 4.4 The current focus does not clarify who is responsible for technical and political approaches at each level

While governments have a leading role in famine prevention (and sometimes, famine creation) in their countries, we have seen that crises frequently involve wider global economic and political systems and actors. It may be more appropriate to speak of the need to make famine prevention a planetary priority and to take a whole‐of‐humanity approach.

Within this broader frame, supporting affected communities at Levels 1 and 2 may be viewed as the purview of local, national, and international humanitarians, whereas Levels 3 and 4 could be seen as more applicable to development and peace actors, as well as policymakers addressing—or contributing to—global inequalities. Yet, even within these levels, recognising different mandates and principles, technical approaches might be implemented by more operational agencies that provide neutral and impartial assistance to support community efforts, whereas political ones may involve civil societies, the UNSC, national governments, advocacy‐oriented organisations, and the ICC and the ICJ. Level 5 issues represent existential threats for the world, requiring the engagement of a wider set of actors. In many cases, these same actors may be involved in various ways at different levels. Achieving agreement on different elements and roles is a critical step and should involve a process of consultation in which affected and at‐risk populations should have a central voice.

### 4.5 Much of the current focus is on actions by governments, donors, and humanitarian, development, and peace entities that underplay the agency of affected communities

It has been shown that in crises, much of the ‘aid’ that affected populations receive comes not from governments or humanitarian organisations, but from neighbours, friends, local organisations, or diasporas. An effective famine prevention framework must embrace these actors and acknowledge and support the central role they play—both in the response and relation to resilience elements.

## 5 CONCLUSION

We have proposed a broad framework for how humanity can better prevent famines. The five levels do not represent separate strategies, but rather a complementary set of actions to be undertaken simultaneously, even if some of their impacts play out on different temporal and spatial scales. But it is also important not to underestimate the difficulty of successfully implementing such a framework. Trends in conflict, climate hazards, economic shocks, and other factors—and the very real challenges of carrying out many of the proposed approaches in practice in recent crises—provide a sobering reminder of how daunting the task would be. Yet it is also possible, looking over time, to recognise the genuine and significant progress that humans have made collectively to prioritise and address the enduring challenge of famine. It is in that spirit that we hope this proposed approach to famine prevention, despite the challenges, will contribute to providing a basis for a planetary framework for the twenty‐first century and beyond.

## ETHICS STATEMENT

This paper reports analysis of primary data. The ethics of data collection and analysis were approved by Tufts University's Institutional Review Board. Persons from whom data were collected gave their free, prior, and informed consent. Their data have been kept confidential and used anonymously.

## FUNDING STATEMENT

The study was supported by the Bureau of Humanitarian Assistance at USAID under the terms of Cooperative Agreement No. 720FDA20CA00065.

## Data Availability

Data sharing not applicable to this article as no datasets were generated or analysed during the current study.^6^

## Endnotes

^1^
The findings in this section draw upon and summarise the content of *Famine Prevention: A Landscape Report* by the cited authors.

^2^
According to the IPC, Phase 5 ‘Famine’ only occurs when: 20 per cent of a population suffers severe acute food insecurity; 30 per cent of children under the age of five are acutely malnourished; and the crude death rate (from hunger malnutrition and disease) is greater than 2 per 10,000 people dying per day. In contrast to the *population‐level* designation of ‘famine’, Phase 5 ‘Catastrophe’ is a *household‐level* description and can be applied to any households experiencing severe acute food insecurity, regardless of the overall percentage of households experiencing the acute food insecurity, the prevalence of acute malnutrition among children, or the crude death rate at the population level.

^3^
Triple nexus efforts may involve local‐level reconciliation and peaceful coexistence, which bring in an element of persuasion and therefore ‘political’ approaches, blurring the distinction with technical efforts. Arguably, this category could have been discussed under political approaches.

^4^
The war in Gaza began after these interviews were conducted but provides an example of the kinds of frustrations that surfaced.

^5^
Responses to existing famines are also critical and would fall into this category, although strictly speaking they would not be famine prevention efforts as they might normally be understood at that point, but rather efforts to prevent the worst effects of famine.
